# Successful Endovascular Treatment of a Traumatic Cervical Arteriovenous Fistula After a Carotid Paraganglioma Resection: A Case Report

**DOI:** 10.7759/cureus.38284

**Published:** 2023-04-29

**Authors:** Alejandro J Quiroz Alfaro, Andrés F Herrera Ortíz, Orlando M Diaz, Joaquin Hidalgo, José A Hakim Tawil, José D Cardona Ortegón, Juan A Mejía

**Affiliations:** 1 Medicine and Health Sciences, Universidad Colegio Mayor de Nuestra Señora del Rosario, Bogotá, COL; 2 Radiology, Fundación Santa Fe de Bogotá, Bogotá, COL; 3 Radiology, Universidad El Bosque, Bogotá, COL; 4 Interventional Neuroradiology, Houston Methodist Neurological Institute, Houston, USA; 5 Neurosurgery, North Mississippi Medical Center, Tupelo, USA; 6 Head and Neck Surgery, Fundación Santa Fe de Bogotá, Bogotá, COL; 7 Interventional Neuroradiology, Fundación Santa Fe de Bogotá, Bogotá, COL

**Keywords:** digital subtraction angiography (dsa), surgical complication, head and neck tumor, carotid paraganglioma, interventional neuroradiology, endovascular interventions, traumatic cervical arteriovenous fistula, onyx embolization, liquid embolic material, arteriovenous (av) fistula

## Abstract

Traumatic cervical arteriovenous fistulas are rare, accounting for only 4% of all arteriovenous fistulas. They can be caused by penetrating, or rarely, blunt trauma, resulting in high-pressure arterial blood draining directly into a vein, decreasing distal perfusion. They are seldom reported as a complication of a carotid paraganglioma surgical resection. Historically, arteriovenous fistulas were treated initially conservatively, after that, surgically; nowadays, endovascular treatment, when feasible, is the preferred method as it offers advantages over surgery. This case report describes a rare traumatic cervical arteriovenous fistula that developed after a carotid paraganglioma resection and was successfully treated using coils and Onyx embolic agent via endovascular embolization. After successful embolization, the patient had a smooth recovery and remained stable. In conclusion, vascular injury seems to be the only constant in all acquired cervical arteriovenous fistulas independent of the trauma mechanism; and endovascular treatment, when feasible, is preferred over surgery as it offers superior advantages.

## Introduction

An arteriovenous fistula (AVF) is an anomalous connection between the arterial and venous systems without an intervening capillary network. AVFs can be caused by iatrogeny, penetrating trauma, or, rarely, blunt trauma, resulting in high-pressure arterial blood draining directly into a vein, decreasing distal perfusion [1-3].

The surgical resection of a carotid paraganglioma can be complicated by dysphagia (10%), facial hemiparesis (6.8%), bleeding (6.8%), hematomas (3.8%), hemiparesis (3.4%), and sinus bradycardia (3.8%); however, an AVF is a rare complication, scarcely reported in the literature [4]. Therefore, we present a case of a patient with a large traumatic cervical arteriovenous fistula (CAVF) secondary to a carotid paraganglioma resection, which was successfully treated endovascularly with coils and Onyx; the therapeutic challenges are described and discussed.

We encourage other authors to report more cases like ours to provide further insight into the epidemiology, management, and outcomes of CAVFs, ultimately contributing to the development of evidence-based guidelines and improving patient care.

## Case presentation

A 54-year-old male presented to the emergency room with painful facial swelling and a pulsatile facial mass. In 2017, the patient had undergone surgery to remove a left carotid paraganglioma requiring the ligation of the middle third of the left external jugular vein. This procedure was complicated by a left vagus nerve injury and ipsilateral vocal cord palsy.

On admission, bulging of the left facial vein was identified during the physical examination, accompanied by signs of venous hypertension on the left facial and left temporal vascular territories, corresponding to the facial mass and swelling reported by the patient; hoarseness secondary to vocal cord palsy was also present. The rest of the examination was unremarkable. 

**Figure 1:**
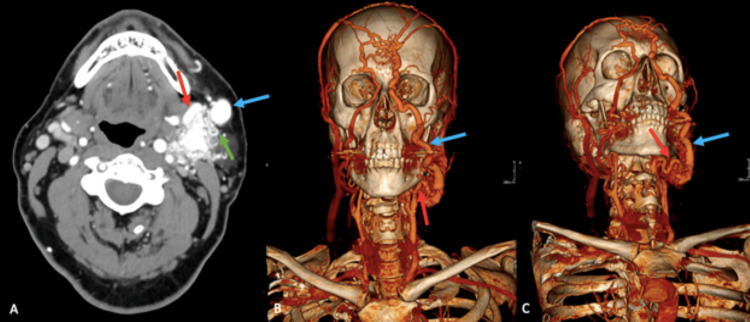
Head and neck computed tomography angiography. The computed tomography angiography images (A, B, C) with volume rendering reconstruction show a large CAVF from the left facial artery (red arrows) to the left facial vein (blue arrows). Notice the prominent abnormal blood vessels, most likely consisting with congested draining veins (green arrow). The middle third of the left external jugular vein is not seen due to ligation during a previous surgical procedure to remove a left carotid paraganglioma. CVAF: cervical arteriovenous fistula

**Figure 2:**
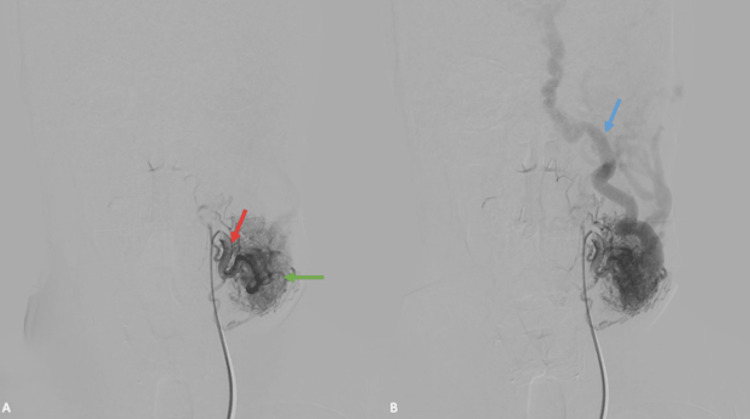
Digital subtraction angiography. Early (A) and late (B) arterial phase selective angiography on an anterior-posterior projection showing the left facial artery (red arrow) and the dilated left facial vein (blue arrow) draining the blood from the CAVF (green arrow). CVAF: cervical arteriovenous fistula

A head and neck computed tomography angiography suggested a large CAVF from the left facial artery to the left facial vein (Figures 1A-1C). Due to the location, feasibility, and high risk of bleeding, the patient underwent a diagnostic and therapeutic angiography confirming the CAVF between the left facial artery and the left facial vein (Figures 2A, 2B).

**Figure 3:**
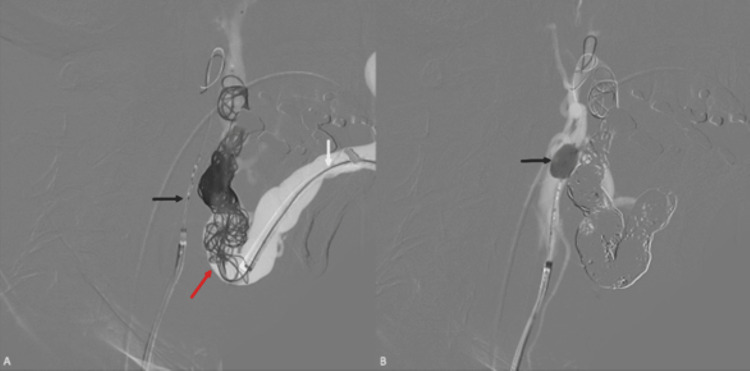
Digital subtraction angiography during embolization procedure. (A) Right-to-left lateral projection of the fluoroscopy-guided retrograde endovascular embolization showing the microcatheter in the dilated left facial vein (white arrow) inserted via direct percutaneous puncture. Notice the embolization agents (coils) occluding a segment of the superior third of the left external jugular vein and its junction with the left facial vein (red arrow), in addition, an ECLIPSE 2L double-lumen 6×7 mm balloon catheter (Montmorency, France: Balt) inserted via a left femoral artery puncture is located in the left external carotid artery (black arrow). (B) The balloon (black arrow) is inflated while the embolization is taking place to help prevent the accidental distal embolization of the facial vein by transiently occluding the arterial flow to the fistulous connection; this allows for a more controlled embolization using a total of 16 coils and the injection of seven vials of Onyx 18. The femoral approach also facilitated a post-procedure contrast injection to verify the occlusion of the fistula.

**Figure 4:**
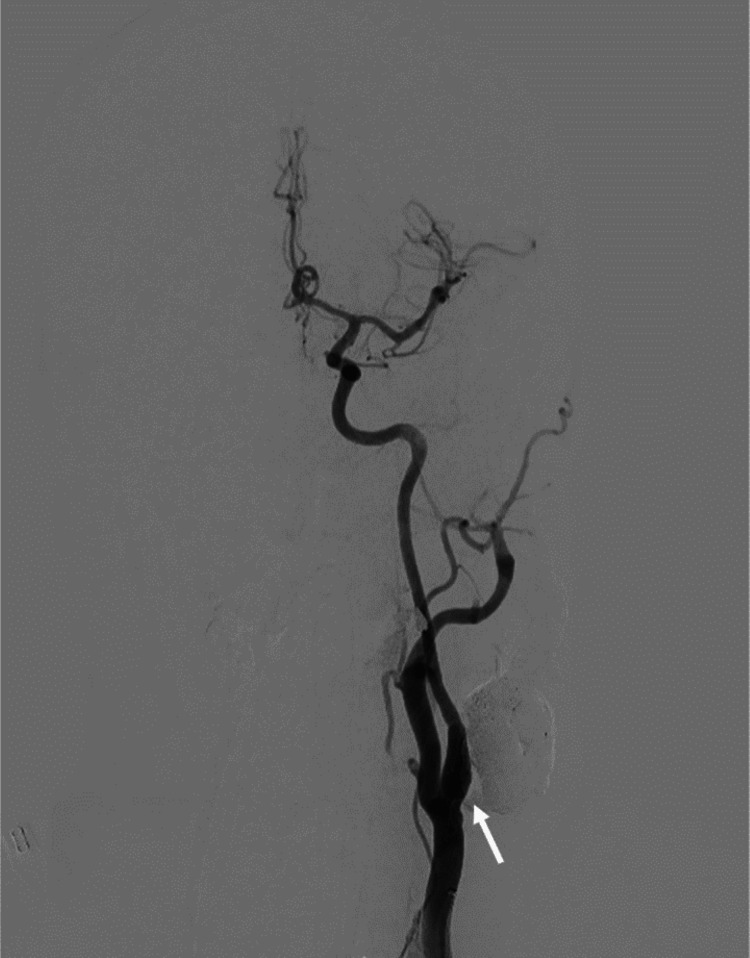
Digital subtraction angiography. An anterior-posterior projection of a left common carotid artery contrast injection showing complete occlusion of the CAVF (white arrow). CVAF: cervical arteriovenous fistula

Therefore, embolization was performed via direct puncture of the dilated left facial vein using a PX SLIM delivery microcatheter (Alameda, CA: Penumbra, Inc.) to deploy 16 detachable coils and inject a total of seven vials of Onyx 18 through an Apollo Onyx delivery microcatheter (Minneapolis, MN: Medtronic) (Figures 3A, 3B). A post-procedure left common carotid artery contrast injection evidenced complete occlusion of the CAVF (Figure 4). There were no complications associated with the procedure. The patient was discharged after a smooth recovery, and a month later, he remained stable.

## Discussion

CAVFs are rare entities; they account for only 4% of all AVFs, and so far, all the information on its epidemiology is comprised of case reports [1]. Although CAVFs are associated with different types of traumas like central line insertions, gunshot wounds, and neck surgical procedures, the vascular injury seems to be the only constant in all acquired cases, independent of the trauma mechanism, even if it is blunt trauma [1,2]. The symptoms caused by AVFs are mainly from retrograde venous congestion (headache, swelling, pulsatile mass, or even glaucoma) or from decreased distal arterial supply (syncope) [5,6]. In our case, the CAVF symptoms were predominantly secondary to increased venous pressure since our patient presented with a prominent and painful facial swelling in the dilated facial vein territory and a pulsatile mass sensation.

There are multiple variations for facial vein drainage; according to Dalip et al. in 2018, the facial vein can drain into the internal jugular vein, into the external jugular vein, or into the subclavian vein [7]. In our case, the patient had a left facial vein that drained into the left external jugular vein, which was ligated during the paraganglioma resection, this alteration of the normal anatomy in our patient could have produced retrograde venous congestion of the facial vein that may have exacerbated the facial swelling and venous hypertension from the CAVF. On the other hand, having the middle third of the external jugular vein ligated helped prevent accidental proximal embolization (to the left brachiocephalic vein and the heart) in our patient.

Historically, AVFs were treated conservatively; later, surgery was the only method of treatment, and nowadays, endovascular treatment, when feasible, is the preferred method as it offers advantages over surgery, like less disability, less bleeding, less post-operative pain, and a faster recovery time [8,9]. Endovascular treatment can be performed by antegrade (arterial approach) or retrograde embolization (venous approach); the latter method is usually preferred when the vessels feeding the fistula are tortuous, difficulting the antegrade access and when the veins that drain the fistula do not contribute to the normal cerebral venous outflow, limiting the risk of a venous infarct [10].

**Figure 5:**
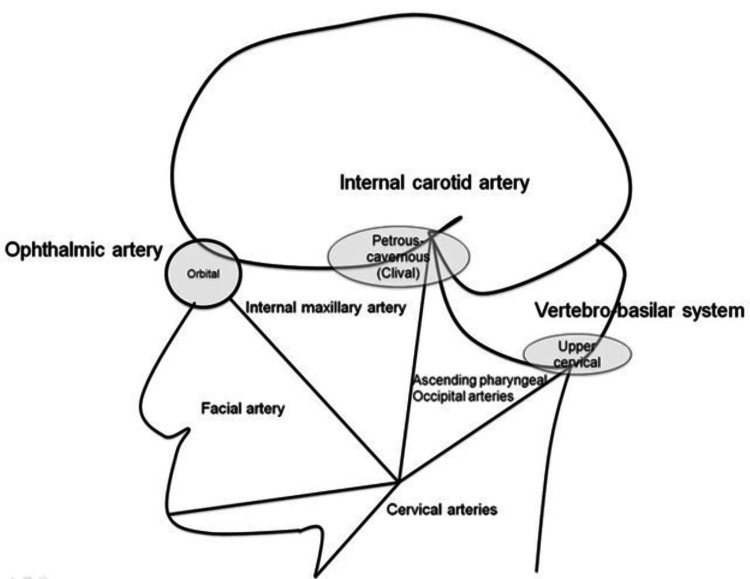
Functional vascular anatomy diagram of the head and neck. Diagram showing the orbital, petrous-cavernous, and upper cervical vascular territories. The image is republished with permission from the American Journal of Neuroradiology (Order license ID 1341028-1).

It is crucial to thoroughly understand the functional vascular anatomy (FVA) of the head and neck to plan an embolization procedure effectively. This concept emphasizes the interconnectedness of adjacent vascular territories (1. orbital territory, 2. petrous-cavernous territory, and 3. upper cervical territory), which can serve as potential collateral pathways in the event of vascular occlusion [11]. For example, if one or several arteries of the petrous-cavernous territory are occluded, the orbital and upper cervical territory could provide collaterals to supply the occluded vessels (Figure 5).

Knowing the anastomoses between the external and internal carotid arteries is relevant to prevent accidental embolization, which can be a complication of transarterial embolization. Loon et al. in 2017 [12] reported a case of an iatrogenic ophthalmic artery occlusion after embolizing the left maxillary artery with microspheres; this is another reason why the transvenous embolization approach was preferred in our patient since the distal end of the facial artery anastomoses with the dorsal nasal artery, a terminal branch of the ophthalmic artery, posing a risk for blindness due to retrograde embolization [11].

## Conclusions

This study highlights the importance of recognizing and adequately managing CAVFs, a rare entity that accounts for only 4% of all AVFs. Understanding the FVA of the head and neck is crucial when planning an effective embolization procedure. Endovascular treatment offers several advantages over surgery, including less disability, bleeding, post-operative pain, and faster recovery time. Our case demonstrates the safety and effectiveness of the transvenous approach for select patients with CAVFs, particularly those at risk of iatrogenic embolization with the transarterial approach. Reporting more cases like this case can provide further insight into the epidemiology, management, and outcomes of CAVFs, ultimately contributing to the development of evidence-based guidelines and improving patient care.
